# Cerebral Small Vessel Disease Biomarkers Detection on MRI-Sensor-Based Image and Deep Learning

**DOI:** 10.3390/s19112573

**Published:** 2019-06-06

**Authors:** Yi-Zeng Hsieh, Yu-Cin Luo, Chen Pan, Mu-Chun Su, Chi-Jen Chen, Kevin Li-Chun Hsieh

**Affiliations:** 1 Department of Electrical Engineering, National Taiwan Ocean University, Keelung 20224, Taiwan; g780455@gmail.com (Y.-C.L.); allen.chen.841202.pan@gmail.com (C.P.); 2 Institute of Food Safety and Risk Management, National Taiwan Ocean University, Keelung 20224, Taiwan; 3 Center of Excellence for Ocean Engineering, National Taiwan Ocean University, Keelung 20224, Taiwan; 4 Department of Computer Science & Information Engineering, National Central University, Taoyuan City 32001, Taiwan; muchun@csie.ncu.edu.tw; 5 Department of Radiology, Shuang Ho Hospital, New Taipei City 23561, Taiwan; 6 Department of Medical Imaging, Taipei Medical University Hospital, Taipei City 110, Taiwan; 7 Translational Imaging Research Center, College of Medicine, Taipei Medical University, Taipei City 110, Taiwan; 8 Department of Radiology, School of Medicine, College of Medicine, Taipei Medical University, Taipei City 110, Taiwan

**Keywords:** MRI-sensor-based image, biomarkers detection, cerebral small vessel disease, deep learning, convolutional neural network, computer-aided diagnosis system

## Abstract

Magnetic resonance imaging (MRI) offers the most detailed brain structure image available today; it can identify tiny lesions or cerebral cortical abnormalities. The primary purpose of the procedure is to confirm whether there is structural variation that causes epilepsy, such as hippocampal sclerotherapy, local cerebral cortical dysplasia, and cavernous hemangioma. Cerebrovascular disease, the second most common factor of death in the world, is also the fourth leading cause of death in Taiwan, with cerebrovascular disease having the highest rate of stroke. Among the most common are large vascular atherosclerotic lesions, small vascular lesions, and cardiac emboli. The purpose of this thesis is to establish a computer-aided diagnosis system based on small blood vessel lesions in MRI images, using the method of Convolutional Neural Network and deep learning to analyze brain vascular occlusion by analyzing brain MRI images. Blocks can help clinicians more quickly determine the probability and severity of stroke in patients. We analyzed MRI data from 50 patients, including 30 patients with stroke, 17 patients with occlusion but no stroke, and 3 patients with dementia. This system mainly helps doctors find out whether there are cerebral small vessel lesions in the brain MRI images, and to output the found results into labeled images. The marked contents include the position coordinates of the small blood vessel blockage, the block range, the area size, and if it may cause a stroke. Finally, all the MRI images of the patient are synthesized, showing a 3D display of the small blood vessels in the brain to assist the doctor in making a diagnosis or to provide accurate lesion location for the patient.

## 1. Introduction

Magnetic resonance imaging (MRI) comprises a magnetic field and radio pulse imaging. Compared to computed tomography (CT), X-ray inspection is a safer method. Although MRI is more expensive than CT, time-consuming (CT is done in tens of seconds and MRI takes tens of minutes), and noisy, the condition of each part of the brain is described in detail. It measures not only the anatomical image of the tissue, but more importantly the various functional images of the organization [1]. The ability to diagnose various tissue and organ functions and metabolic disorders is the biggest feature of MRI. MRI has a higher resolution of soft tissue. Magnetic resonance imaging has been widely used as a medical imaging approach, and MRI is a safe way to detect the diseases of life. Although MRI is not dangerous radiation imaging, its long processing time is inconvenient to patients in time-depended diseases, like strokes. In addition, it finds lesions earlier. For the head and brain, MRI can help detect brain tumors, vascular wall protrusions (hemangioma), blood clots in the brain, destruction of nerve fibers due to multiple sclerosis, and other forms of brain damage (like brain injury caused by stroke) [2]. Cerebrovascular disease, which is the most common brain stroke, is also the second most common cause of death in the world [3]. The symptoms of cranial nerve defects caused by stroke are very diverse, with different clinical manifestations of brain damage in different parts. In addition to the common half body squat, half body feeling numb, mouth slanting, and unclear speech, it also includes sudden changes in consciousness, mental decline, poor vision and visual field defects, diplopia, deafness, and lack of orientation. Cerebral small vessel disease may cause patients to have difficulty breathing, or even lead to patients’ death [4]. There are three common causes of stroke in the brain. The most common and fatal is small blood vessel lesions in the brain. It is a cerebral infarction caused by a single small blood vessel obstruction. The cerebral small vessel in a MRI image is less than 1.5 cm. It is not easy to see, and must be determined by the careful examination of the clinician; the whole process takes a considerable amount of time and effort to complete.

Therefore, the purpose of this paper was to use the huge amount of MRI image data created by Taipei Medical University-Shuang Ho Hospital, Ministry of Health and Welfare, New Taipei, Taiwan, based on the method of deep learning and use of a convolutional neural network, to discover clear features that identify the blocks of cerebrovascular disease. Convolutional neural networks (CNNs) are inclined to be susceptive to the alterations in obtaining MRI protocols. Our experiment of MRI protocol is T1 weighting, turbo spin echo (TSE) imaging, 3.4 mm length of slice, and 1 mm of slice gap. The features extracted from the MRI images of multiple slices can be used to calculate the distribution location and area of the lesion block, establish a 3D model to simulate the entire brain lesion location, and quickly find the problem, thereby assisting the physician in the diagnosis of the auxiliary system.

## 2. Related Works

In recent years, there has been a growing use of deep learning to solve medical problems, such as detecting lung symptoms, brain tumor location, etc. [5,6], and such studies have also been shown to help medically. In view of this, regarding diagnostics and assistance, we have begun to make annotations in the medical image database that can be used for the MRI images created by Taipei Medical University-Shuang Ho Hospital, Ministry of Health and Welfare, New Taipei, Taiwan, as such [7]. The database mainly contains the part of the stroke, the age layer is mostly middle-aged patients, 50 patients have a total of 1000 data, and brain stroke caused by cerebral small vessel disease is marked. The data content provides information on the location, extent, and symptoms of the lesion. The database is used as a label to identify if there is a stroke. In order to obtain more accurate data, our proposed method could help doctors detect cerebral small vessels from the MRI imaging, and doctors can determine if the vessel area position influences the patient’s life clinically or not.

### Convolutional Neural Network

Deep neural networks have been widely used to achieve the latest results in various categories, including face detection, image recognition, and target detection [8,9,10]. The convolutional neural network is a deep learning method; this method has achieved very good results in solving image recognition and classification. The AlexNet [11] that was based on convolutional neural network running in GPU won the ImageNet Large Scale Visual Recognition Challenge in 2012. However, our proposed method is not designed on GPU because we wanted to utilize computer resources as little as possible. Therefore, our proposed framework of CNN was a 7-layers structure at most and run in CPU, and the performance is much higher accuracy than other methods. The use of deep neural networks for image recognition is now mainstream, and with the popularity of deep networks, there are many different CNN architectures such as AlexNet [11], ZFnet [12], GoogLeNet [13], and ResNet [14]. These architectures are based on deep learning, and there are effectively applied to the classification of objects [15,16,17,18] in image. Most of them are using Gradient-weighted Class Activation Mapping (Grad-CAM) to calculate the error signal from developing the gradients with objective function. Reference [19] proposed a novel method called “class-selective relevance mapping” (CRM) which improved the localizing and visualizing discriminative regions of interest (ROI) within a medical image. In view of this, we also intended to use depth. The neural network detects the area of cerebral small vessel lesions in the MRI image of the brain and seeks to find the features of the CNN structure and classify the ranking of the stroke and the location of the block [20,21].

## 3. The Proposed Method

### 3.1. Data Preprocessing

**Figure 1:**
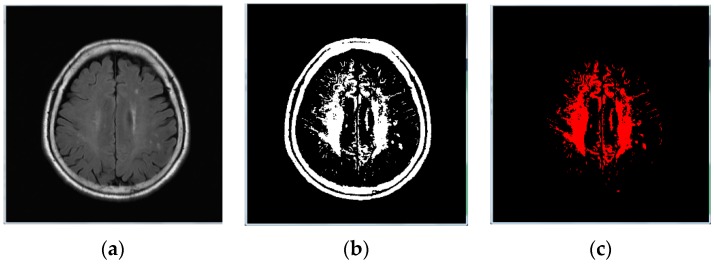

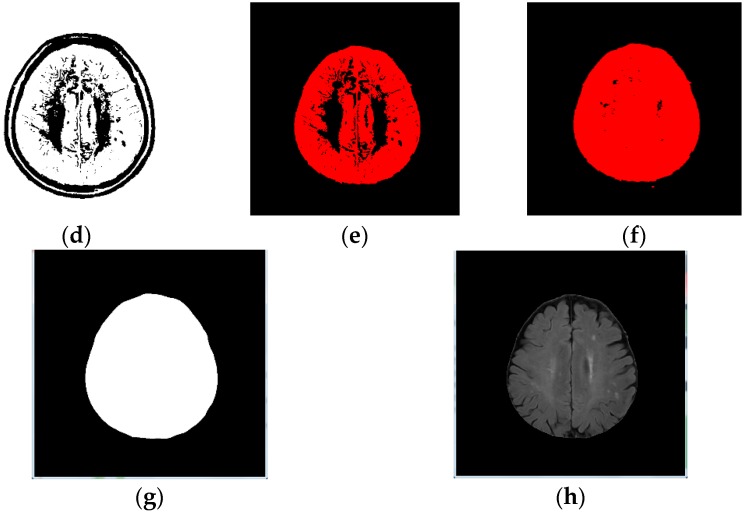
(**a**) Original brain image; (**b**) image binarization; (**c**) removed head shell; (**d**) image binarization (reverse); (**e**) removal of the head shell; (**f**) steps 2 and 4 added; (**g**) mask image; (**h**) result image (header).

Before performing the deep learning detection method, we first processed the image to remove any unnecessary parts of the image. In a brain MRI image (Figure 1b), a complete brain cross can be seen. The image was of the facet, but what we wanted to detect was the block of the brain that had blocked or broken small blood vessels. Our proposed method calculated the area size of each of the connected components from the binary image, and then the maximum size of connected components was removed. Therefore, in Figure 1b, we removed the part of the head shell and kept only the whole brain part, using the steps as follows:

**Step** **1:** Image binarization: We made sure the cranium (head shell) with the maximum region in image (Figure 1b);
**Step** **2:** Remove the head shell: After image binarization, the connected component method [22] was adopted to identify the area size of each block (Figure 1b). Then, the cranium was removed by detecting the maximum area (Figure 1c);
**Step** **3:** Image inverse binarization: The image from step 1 was adopted the inversed-binarization method in order to obtain the cerebrum region (Figure 1d,e);
**Step** **4:** Identify the cerebrum region: The cerebrum region was obtained from step 3 and then compared with the region obtained by step 2. The union of the two step regions was calculated to identify the cerebrum region (Figure 1f);
**Step** **5:** Perform median filtering to remove noise (Figure 1g); and
**Step** **6:** Calculate the actual size and position of the brain (Figure 1h).

Through the above steps, we can remove the non-brain part of the brain MRI image leaving only the brain part. There are two important reasons for this. The first is because the lesion of the small blood vessels does not occur on the bones of the head, but only in the brain; another reason is because the size, location, and shape of the small blood vessel lesions differ, and it is impossible to calculate the precise position of small vessel lesions. The block with cerebral small vessel disease accounts for only a small part of the overall brain image. Because the doctor must detect the cerebral small vessel position, we must locate where the vessel position is in the brain. Additionally, the small vessels cannot occur in non-cerebral areas like the cranium, so our proposed method deleted the non-cerebral area.

### 3.2. Training Model

**Figure 2:**
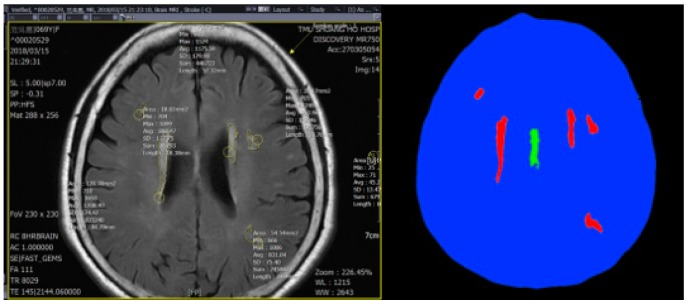
(**Left**) is an image of the area of the cerebral small vessel lesion by the physician, and (**right**) the image marked by the physician as a ground-truth.

We used the data set provided by the Taipei Medical University-Shuang Ho Hospital, Ministry of Health and Welfare, New Taipei, Taiwan. There were data on 30 patients. To avoid over-estimating, we separated the 30 patients into three parts: 10 patients for training, 10 patients for verification, and 10 for testing. There were a total of 616 images in which each image size was 512 × 512 pixels, of which 205 images were used as training sets and 205 images were used as verification sets. The remaining 206 images were used as test sets. Before training, we first made the image marked by the doctor into the ground-truth image for training (Figure 2), and drew four labels for each image: The image of the non-brain block (black part), the normal part of the brain (green part), the normal brain position (blue position), and the cerebrovascular disease block (red part). The ground-truth image was used as training data to perform supervised training [23]. We retrieved the MRI imaging part from the other non-imaging part of raw data.

**Figure 3:**
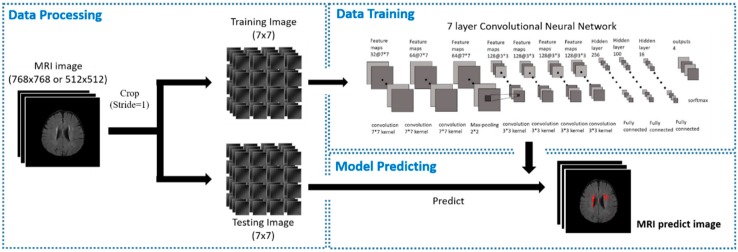
The proposed method architecture.

According to the above classification, we can use CNN to find the characteristics of each category, and the images used for testing can also be used to find the areas of small blood vessel lesions through the identified features. Unlike other typical training methods, we looked for the part of the brain MRI image that contained cerebral small vessel lesions, as stated in the previous section, and for each image, the size, shape, location, and area of the lesion. The difference between the number of blocks was very large, and most of the lesions only accounted for a small part of the overall image, so we could not directly use the whole image for the training model, unlike other image recognition methods, because it hindered identification. Therefore, our proposed method (Figure 3), according to deep learning, can detect the small vessels directly without the morphology process.

**Figure 4:**
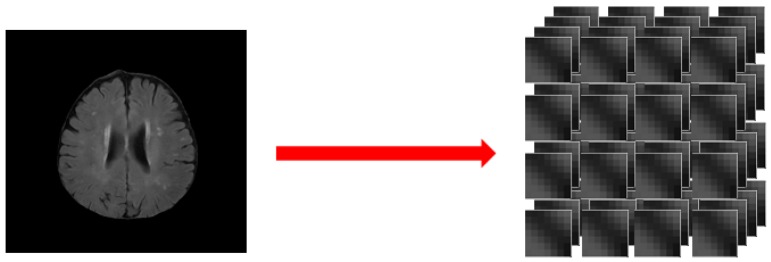
Brain MRI segmentation based on patch convolutional neural network (CNN) method, splitting the image into patches of size 7 × 7 pixels.

In order to identify the small vessel lesions regions, we used the MRI segmentation of the brain based on the patch CNN method [24], and divided the MRI image of the brain through the removal of the head shell into several pieces; each block size was 7 × 7 pixels. Our MRI imaging was all with 2D images and 7 × 7 patches were the best detection results in our experiment. These images, which were divided into blocks, can be combined into the size of the original image. These small images are called patches to ensure that we can get complete and accurate images (Figure 4).

**Figure 5:**
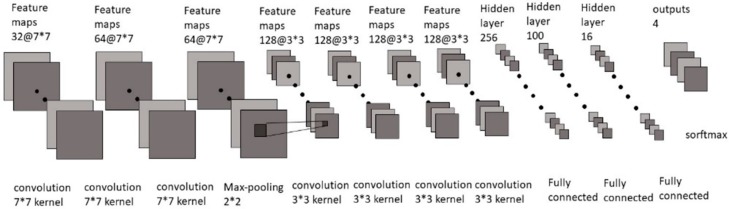
CNN model architecture.

**Figure 6:**
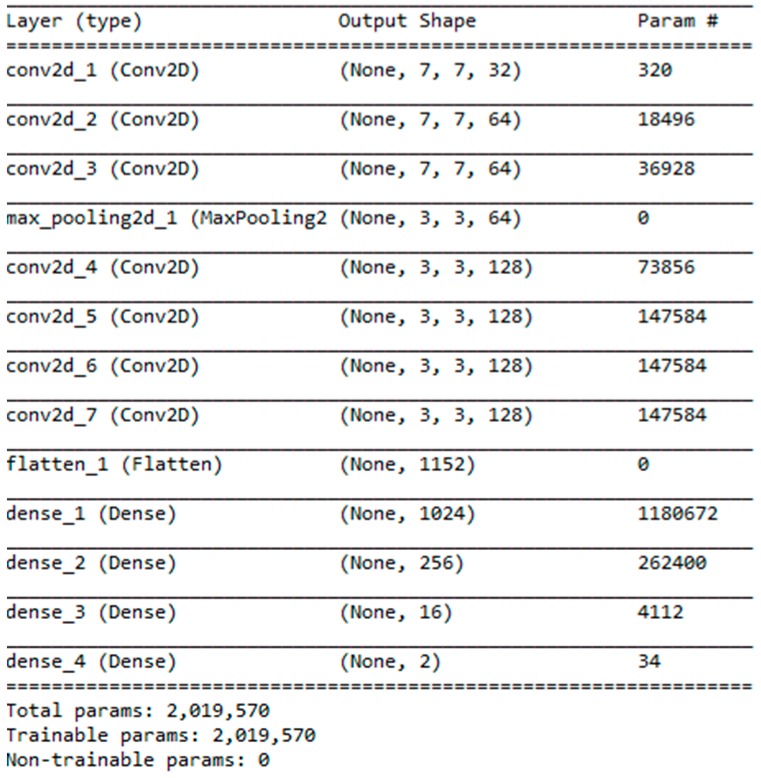
Our proposed CNN architecture.

Next, we sent the segmented image into our 7-layers CNN model as training (Figure 5), and finally used a softmax function as a classifier to divide the results into four categories. The structure of each layer (Figure 6) includes where the filter extracts the features for each image, and continues to find other features in the next layer of filters, while maxpooling makes the image smaller, which helps reduce the excessive repetition of training. Regarding features, we finally found the weight and bias value of each feature through several fully connected layers, and displayed the result of the classification as a decimal point (percentage) through softmax. We used a NVIDIA/GTX1060 3GB graphics card and tensorflow/keras tools as training programs to build the model architecture and then train.

## 4. Experiment Results

**Figure 7:**
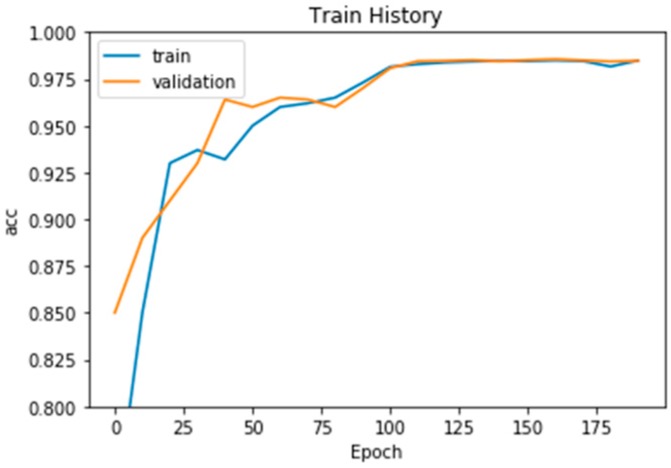
CNN model training results.

After the above image preprocessing and segmentation method, we sent the data into our constructed CNN model; the final training result was 0.9857 (98.57%), and the image result used for verification was 0.9852 (98.52%). However, our proposed method was not designed on GPU, because we wanted to utilize computer resources as little as possible. Therefore, our proposed framework of CNN is a 7-layers structure at most and run in CPU. Therefore, the architecture of our proposed CNN model was based on our computer resources. The final data in Figure 7 indicate that our training results were useful for detecting block cerebral small vessel disease.

**Figure 8:**
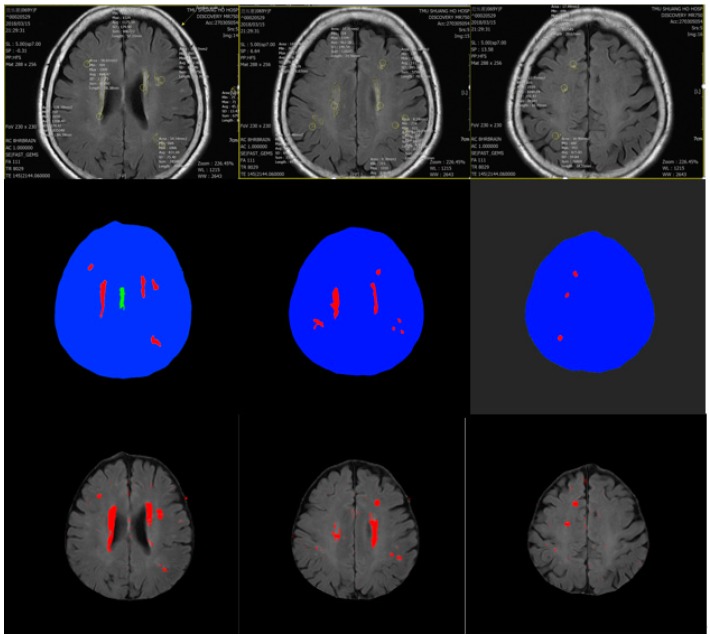
(**Top**) image marked by the physician, (**middle**) ground-truth drawn according to the position marked by the physician, (**bottom**) image output by the CNN model.

Taipei Medical University-Shuang Ho Hospital provided the MicroDicoM dicom Viewer to doctors to help clinical decisions. Most of the brain MRI images have been mentioned before, and the lesion-containing blocks account for a very small overall image. The position of cerebral small vessels affects the patient’s life or other activities, and therefore the position of cerebral small vessel must be detected. There were lesions of cerebral small blood vessels, and the number of lesions was much smaller than the other three categories (non-brain area, central brain area, and brain area). It is possible that the error rate of the diseased block was high, but in regard to the overall training result, the impact was very small (the amount of lesions was small, and the influence of other types of data was relatively small), so in order to actually confirm that our output was in line with the doctor’s label, we finally output the results of the training model on the original MRI image (Figure 8). It can be seen from the image that our results were quite consistent with the results marked by the physician. The red mark in the figure is the part of the small blood vessel lesion, according to Figure 7. The accuracy from left to right is 98.83%, 96.23%, and 97.45%, respectively. According to Figure 8, our model could indeed mark the position marked by the doctor. Larger lesions have better output, and some smaller ones, although not obvious, can be expressed. Therefore, we have determined that the training of the model was successful, and the images output by the model can be helpful when assisting clinicians in diagnosis.

### Comparison with Other Training Models

**Figure 9:**
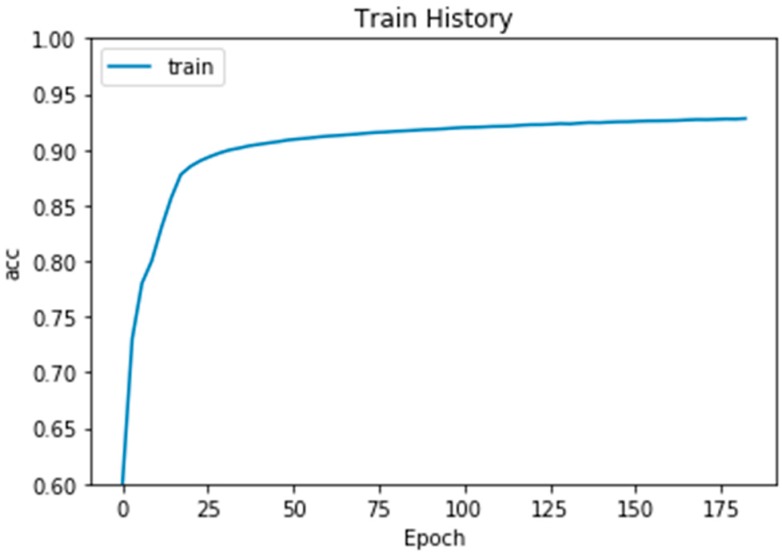
Multilayer perceptron (MLP) model.

Our experiment split the number of training data, validation data, and testing data into 205, 205, and 206 images, respectively, and then our experiment adopted the 10-fold cross validation to avoid overfitting. The MRI images were provided by the Taipei Medical University-Shuang Ho Hospital. Because the model designed by a deep neural network can be used to assist physicians in diagnosing cerebral small vessel disease, we also used other models for comparison. We used a multilayer perceptron (MLP) model as a comparison with our model (Figure 9) and the training iteration number was the same as with the trained CNN model (ex.180). The results of the training indicated that an accuracy rate of nearly 97.5% can be achieved, which is not much different from our training accuracy rate of 98.52%, and even tends to converge in the earlier training times.

Our method is not like the “You Only Look Once” (YOLO) algorithm [25,26,27] that detects the object boundary by the intersection of the union (IoU). Additionally, we adopted the 10-folds to measure the mAP(mean Average Precision) for the training data set and the testing data set, and also to compare with YOLO1 [25], YOLO2 [26], and YOLO3 [27]. We also compared with different algorithms according to F1 score (F-score, F-measure), precision rate, recall rate, True Positive (TP), False Positive (FP), False Negative (FN) and True Negative (TN) (Table 1, Table 2, Table 3, Table 4, Table 5, Table 6, Table 7, Table 8, Table 9 and Table 10).

(1) $$F1\;\mathrm{Score}=\frac{2}{\frac{1}{recall}+\frac{1}{precision}}$$

(2) $$\mathrm{Precision}=\frac{TP}{\left(TP+FP\right)}$$

(3) $$\mathrm{Recall}=\frac{FN}{\left(TP+FN\right)}$$

**Figure 10:**
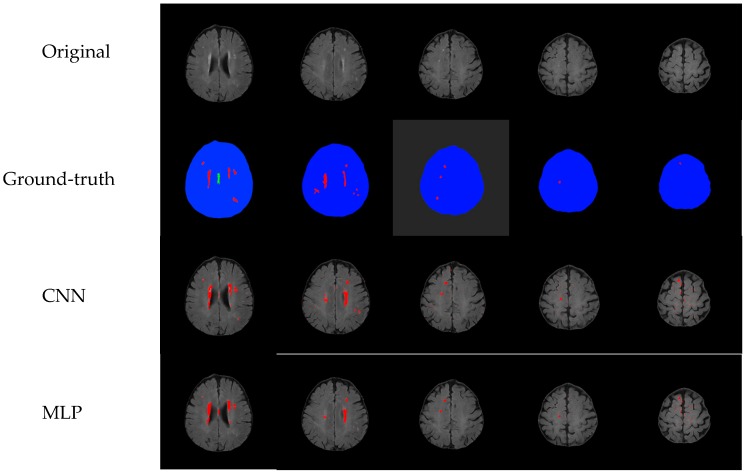
Comparison of CNN and MLP models.

Our proposed method can help doctors to detect cerebral small vessels from the MRI imaging, and doctors can determine if the vessel area position influences the patient’s life clinically or not. The accuracy of the training alone does not represent the quality of the model. Therefore, we also compared the images output of the MLP model with the CNN model. It can be seen from Figure 10 that the MLP model can also mark the lesion position, but the part of the normal block in the middle of the brain is not small vessel lesions (Figure 9), and the lesions with smaller blocks cannot be found (Figure 10). So, we can be sure that the image output by the CNN model is better than the MLP model.

## 5. Conclusions and Future Work

Through the model structure of the deep neural network CNN, the model we designed was very good in relation to the output image. If the block was too small, it is difficult to detect the lesion. However, this study demonstrated that the approximate block position also showed that by combining the removal of the head bone with the MRI segmentation of the brain based on the patch CNN method, the training of the CNN model can improve the accuracy of detecting lesion. Admittedly, this study still has many areas that can be improved. For each different MRI image of the brain, we removed the part of the head bone according to its characteristics. This part still has to be done manually by the method based on patch CNN brain MRI segmentation. Although it can be ensured that the lesions are also detected, the circle will be slightly larger than the image marked by the doctor (about 2 pixels), and cannot completely replace the image that the doctor is paying attention to. However, for the association and grading of stroke caused by cerebral small vessel disease, some data available today are not relevant. It is important to help the doctor diagnose the level of stroke by observing the small vessel lesions region and position. In the future, these data will be used for deep neural network training, and the model training for improved design will achieve stability and good accuracy. We can display the lesion block, stroke, or stroke with the stroke level to form a 3D image of each brain MRI image to assist physicians in their diagnosis.

## Figures and Tables

**Table 1:** Training mAP of our proposed model.

Training mAP	Cerebral Small Vessel	Non Cerebral Small Vessel
Cerebral small vessel	94/95 (TP)	2/110 (FP)
Non cerebral small vessel	1/95 (FN)	108/110 (TN)

**Table 2:** Testing mAP of our proposed model.

Testing mAP	Cerebral Small Vessel	Non Cerebral Small Vessel
Cerebral small vessel	84/86 (TP)	5/120 (FP)
Non cerebral small vessel	2/86 (FN)	115/120 (TN)

**Table 3:** Training mAP of YOLO1 model.

Training mAP	Cerebral Small Vessel	Non Cerebral Small Vessel
Cerebral small vessel	90/95 (TP)	6/110 (FP)
Non cerebral small vessel	5/95 (FN)	104/110 (TN)

**Table 4:** Testing mAP of YOLO1 model.

Testing mAP	Cerebral Small Vessel	Non Cerebral Small Vessel
Cerebral small vessel	80/86 (TP)	8/120 (FP)
Non cerebral small vessel	6/86 (FN)	112/120 (TN)

**Table 5:** Training mAP of YOLO2 model.

Training mAP	Cerebral Small Vessel	Non Cerebral Small Vessel
Cerebral small vessel	91/95 (TP)	7/110 (FP)
Non cerebral small vessel	4/95 (FN)	103/110 (TN)

**Table 6:** Testing mAP of YOLO2 model.

Testing mAP	Cerebral Small Vessel	Non Cerebral Small Vessel
Cerebral small vessel	80/86 (TP)	8/120 (FP)
Non cerebral small vessel	6/86 (FN)	112/120 (TN)

**Table 7:** Training mAP of YOLO3 model.

Training mAP	Cerebral Small Vessel	Non Cerebral Small Vessel
Cerebral small vessel	89/95 (TP)	9/110 (FP)
Non cerebral small vessel	6/95 (FN)	101/110 (TN)

**Table 8:** Testing mAP of YOLO3 model.

Testing mAP	Cerebral Small Vessel	Non Cerebral Small Vessel
Cerebral small vessel	79/86 (TP)	10/120 (FP)
Non cerebral small vessel	7/86 (FN)	110/120 (TN)

**Table 9:** F1-score, precision rate, recall rate of training data.

	Our Method	YOLO1	YOLO2	YOLO3
F1-score	0.020829346	0.080591758	0.080591758	0.118198648
Precision rate	0.981956315	0.937704918	0.937704918	0.919680601
Recall rate	0.010526316	0.042105263	0.042105263	0.063157895

**Table 10:** F1-score, precision rate, recall rate of testing data.

	Our Method	YOLO1	YOLO2	YOLO3
F1-score	0.045410519	0.129827978	0.129827978	0.149516707
Precision rate	0.959086584	0.933125972	0.933125972	0.916827853
Recall rate	0.023255814	0.069767442	0.069767442	0.081395349
